# Anthracene-1,4,9,10-tetra­one

**DOI:** 10.1107/S1600536813026342

**Published:** 2013-10-02

**Authors:** Chitoshi Kitamura, Takeshi Kawase

**Affiliations:** a Department of Materials Science, School of Engineering, The University of Shiga Prefecture, 2500 Hassaka-cho, Hikone, Shiga 522-8533, Japan; b Department of Materials Science and Chemistry, Graduate School of Engineering, University of Hyogo, 2167 Shosha, Himeji, Hyogo 671-2280, Japan

## Abstract

The asymmetric unit of the title compound, C_14_H_6_O_4_, contains three independent mol­ecules (*A*, *B* and *C*). In mol­ecule *C*, there are two disordered sets of two carbonyl O atoms [occupancies = 0.643 (11) and 0.357 (11)]. All three mol­ecules are non-planar due to repulsion between two O atoms in *peri* positions on the anthracene ring, showing a slight difference in deviation of the carbonyl O atoms. The intra­molecular distances between the two nearest O atoms are in the range of 2.685 (10)–2.766 (10) Å. In the crystal, mol­ecules are linked by C—H⋯O and π–π [centroid–centroid distances = 3.615 (2), 3.844 (2) and 3.921 (2) Å] inter­actions, which lead to the formation of a herringbone-like arrangement.

## Related literature

For the synthesis of the title compound, see: Yoshino *et al.* (1981 ▶). For applications of 1,4,9,10-anthracene­tetra­one (quinizarindi­quinone) derivatives, see: Isikli & Díaz (2012 ▶); Adeva *et al.* (1997 ▶); Jin *et al.* (1998 ▶).
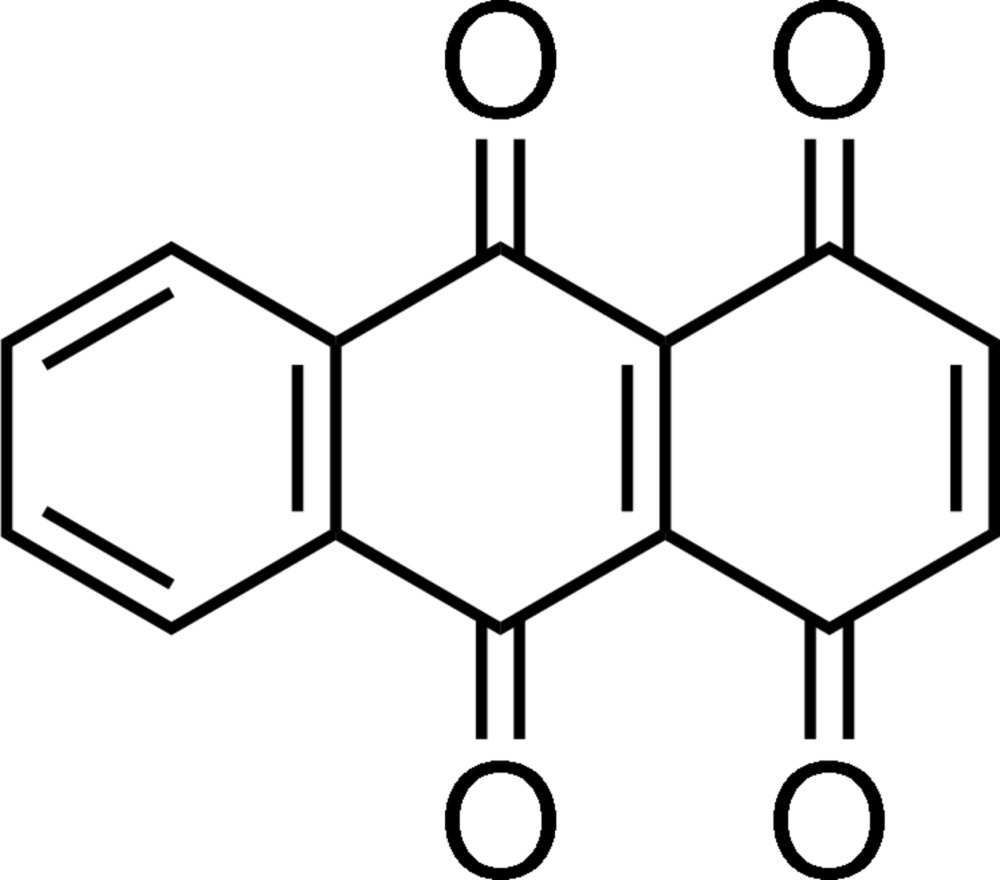


## Experimental

### 

#### Crystal data

C_14_H_6_O_4_
*M* *_r_* = 238.19
Monoclinic, 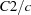

*a* = 39.450 (4) Å
*b* = 5.4465 (5) Å
*c* = 32.787 (3) Å
β = 119.185 (9)°
*V* = 6150.4 (11) Å^3^
*Z* = 24
Mo *K*α radiation
μ = 0.12 mm^−1^
*T* = 223 K
0.55 × 0.08 × 0.04 mm

#### Data collection

Rigaku R-AXIS RAPID diffractometer
27613 measured reflections
7016 independent reflections
3239 reflections with *I* > 2σ(*I*)
*R*_int_ = 0.126

#### Refinement

*R*[*F* ^2^ > 2σ(*F* ^2^)] = 0.070
*wR*(*F* ^2^) = 0.204
*S* = 0.99
7016 reflections
506 parameters
4 restraints
H-atom parameters constrained
Δρ_max_ = 0.43 e Å^−3^
Δρ_min_ = −0.43 e Å^−3^

### 

Data collection: *PROCESS-AUTO* (Rigaku, 1998 ▶); cell refinement: *PROCESS-AUTO*; data reduction: *PROCESS-AUTO*; program(s) used to solve structure: *SIR2004* (Burla *et al.*, 2005 ▶); program(s) used to refine structure: *SHELXL2013* (Sheldrick, 2008 ▶); molecular graphics: *ORTEP-3 for Windows* (Farrugia, 2012 ▶) and *Mercury* (Macrae *et al.*, 2008 ▶); software used to prepare material for publication: *WinGX* (Farrugia, 2012 ▶).

## Supplementary Material

Crystal structure: contains datablock(s) global, I. DOI: 10.1107/S1600536813026342/vm2198sup1.cif

Structure factors: contains datablock(s) I. DOI: 10.1107/S1600536813026342/vm2198Isup2.hkl

Supplementary material file. DOI: 10.1107/S1600536813026342/vm2198Isup3.cml

Additional supplementary materials:  crystallographic information; 3D view; checkCIF report

## Figures and Tables

**Table 1:** Hydrogen-bond geometry (Å, °)

*D*—H⋯*A*	*D*—H	H⋯*A*	*D*⋯*A*	*D*—H⋯*A*
C4*C*—H4*C*⋯O1*A* ^i^	0.94	2.45	3.373 (5)	169
C5*C*—H5*C*⋯O2*A* ^i^	0.94	2.33	3.112 (5)	140
C11*A*—H11*A*⋯O1*C* ^ii^	0.94	2.55	3.262 (7)	133
C11*C*—H11*C*⋯O3*B* ^iii^	0.94	2.52	3.113 (4)	121
C12*C*—H12*C*⋯O3*B* ^iii^	0.94	2.49	3.095 (5)	122

Symmetry codes: (i) 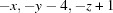; (ii) 
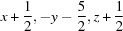
; (iii) 
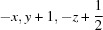
.

**Fig. 1.**
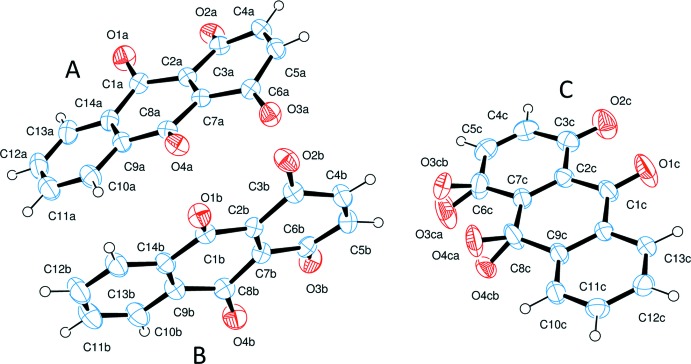
The molecular structure of the title compound, showing the atomic numbering and 40% probability displacement ellipsoids.

**Fig. 2.**
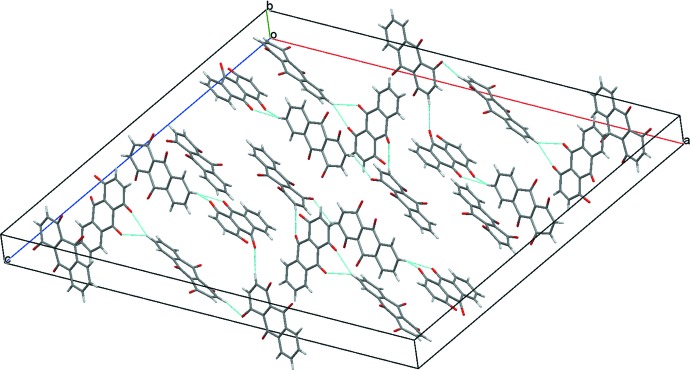
The packing diagram of the title compound. C—H···O interactions are shown as blue lines.

## supplementary crystallographic information

### 1. Comment

1,4,9,10-Anthracenetetraone (quinizarindiquinone) derivatives are important electrode matetrials (Isikli & Díaz, 2012) as well as effective dienophiles for Diels-Alder reaction (Adeva *et al.*, 1997). Furthermore, the antitumor activity was also investigated (Jin *et al.*, 1998). We have recently investigated the solid-state electrochemical properties of quinone compounds. To elucidate the molecular and packing structures, the crystallographic study of the title compound was carried out. The title compound (I) was synthesized as previously reported (Yoshino *et al.* (1981)). The title compound, C_14_H_6_O_4_, crystallizes with three molecules in the asymmetric unit, as shown in components A, B, and C (Fig. 1). All the molecules are not planar because of the mutual repulsion of two oxygen atoms on the same side on the anthracene ring. In the component C, there are two disordered sets of oxygen atoms (O3CA/O4CA with occupancy 0.643 (11) and O3CB/O4CB with occupancy 0.357 (11)) although the other components are not disordered. The intramolecular distances between two nearest oxygen atoms are in the range of 2.685 (10)–2.766 (10) Å (O1A···O2A, 2.710 (6) Å; O3A···O4A, 2.721 (5) Å; O1B···O2B, 2.726 (5) Å; O3B···O4B, 2.708 (6) Å; O1C···O2C, 2.674 (5) Å; O3CA···O4CA, 2.766 (10) Å; O3CB···O4CB, 2.685 (10) Å), all of which are shorter than the sum of van der Waals radii of two oxygen atoms. As shown in Fig. 2, in the crystal, molecules are linked by many C—H···O interactions (Table 1). The crystal packing is also controlled by π-π interactions.

### 2. Experimental

The title compound was prepared according to the literature procedure (Yoshino *et al.*, 1981). Single crystals suitable for X-ray analysis were obtained by recrystallization from ethyl acetate.

### 3. Refinement

All the H atoms were positioned geometrically and refined using a riding model with C—H = 0.94 Å and *U*_iso_(H) = 1.2*U*_eq_(C) for aromatic C—H. In molecule C, two O atoms on the *peri* positions of the anthracene ring are disordered over two positions with site occupancies of 0.643 (11) and 0.357 (11). During the refinement, the two O atoms were refined with a restrained C—O distance of 1.21 Å.

### Crystal data

**Table:**

C_14_H_6_O_4_	*F*(000) = 2928
*M**_r_* = 238.19	*D*_x_ = 1.543 Mg m^−^^3^
Monoclinic, *C*2/*c*	Mo *K*α radiation, λ = 0.71073 Å
Hall symbol: -C 2yc	Cell parameters from 8613 reflections
*a* = 39.450 (4) Å	θ = 3.1–27.5°
*b* = 5.4465 (5) Å	µ = 0.12 mm^−^^1^
*c* = 32.787 (3) Å	*T* = 223 K
β = 119.185 (9)°	Needle, brown
*V* = 6150.4 (11) Å^3^	0.55 × 0.08 × 0.04 mm
*Z* = 24	

### Data collection

**Table:**

Rigaku R-AXIS RAPID diffractometer	3239 reflections with *I* > 2σ(*I*)
Radiation source: fine-focus sealed x-ray tube	*R*_int_ = 0.126
Graphite monochromator	θ_max_ = 27.5°, θ_min_ = 3.1°
Detector resolution: 10 pixels mm^-1^	*h* = −50→50
φ and ω scans	*k* = −7→6
27613 measured reflections	*l* = −42→41
7016 independent reflections	

### Refinement

**Table:**

Refinement on *F*^2^	Primary atom site location: structure-invariant direct methods
Least-squares matrix: full	Secondary atom site location: difference Fourier map
*R*[*F*^2^ > 2σ(*F*^2^)] = 0.070	Hydrogen site location: inferred from neighbouring sites
*wR*(*F*^2^) = 0.204	H-atom parameters constrained
*S* = 0.99	*w* = 1/[σ^2^(*F*_o_^2^) + (0.087*P*)^2^] where *P* = (*F*_o_^2^ + 2*F*_c_^2^)/3
7016 reflections	(Δ/σ)_max_ < 0.001
506 parameters	Δρ_max_ = 0.43 e Å^−^^3^
4 restraints	Δρ_min_ = −0.43 e Å^−^^3^
0 constraints	

### Special details

**Table:**

Geometry. All e.s.d.'s (except the e.s.d. in the dihedral angle between two l.s. planes) are estimated using the full covariance matrix. The cell e.s.d.'s are taken into account individually in the estimation of e.s.d.'s in distances, angles and torsion angles; correlations between e.s.d.'s in cell parameters are only used when they are defined by crystal symmetry. An approximate (isotropic) treatment of cell e.s.d.'s is used for estimating e.s.d.'s involving l.s. planes.

### Fractional atomic coordinates and isotropic or equivalent isotropic displacement parameters (Å^2^)

**Table:**

	*x*	*y*	*z*	*U*_iso_*/*U*_eq_	Occ. (<1)
O1A	0.14529 (8)	−1.7063 (5)	0.68248 (9)	0.0598 (7)	
O2A	0.07223 (8)	−1.8371 (4)	0.61754 (9)	0.0536 (7)	
O3A	0.05999 (8)	−1.0566 (4)	0.51908 (8)	0.0518 (7)	
O4A	0.13395 (7)	−0.9228 (4)	0.58144 (8)	0.0460 (6)	
C1A	0.14361 (11)	−1.5575 (6)	0.65379 (11)	0.0412 (8)	
C2A	0.10561 (10)	−1.4833 (5)	0.61297 (11)	0.0361 (8)	
C3A	0.07008 (11)	−1.6314 (6)	0.60195 (11)	0.0401 (8)	
C4A	0.03261 (11)	−1.5208 (6)	0.57124 (12)	0.0453 (9)	
H4A	0.01	−1.5935	0.5685	0.054*	
C5A	0.02961 (11)	−1.3194 (6)	0.54695 (12)	0.0442 (8)	
H5A	0.005	−1.2492	0.5282	0.053*	
C6A	0.06389 (10)	−1.2044 (5)	0.54878 (11)	0.0378 (8)	
C7A	0.10251 (10)	−1.2810 (5)	0.58779 (11)	0.0347 (7)	
C8A	0.13729 (10)	−1.1234 (6)	0.59924 (11)	0.0366 (8)	
C9A	0.17549 (10)	−1.2191 (6)	0.63406 (11)	0.0392 (8)	
C10A	0.20923 (11)	−1.0931 (6)	0.64241 (14)	0.0503 (9)	
H10A	0.2073	−0.9471	0.6261	0.06*	
C11A	0.24506 (12)	−1.1818 (7)	0.67426 (14)	0.0569 (10)	
H11A	0.2676	−1.0967	0.6796	0.068*	
C12A	0.24817 (13)	−1.3966 (8)	0.69860 (14)	0.0613 (11)	
H12A	0.2728	−1.4593	0.7197	0.074*	
C13A	0.21521 (12)	−1.5182 (7)	0.69196 (13)	0.0531 (10)	
H13A	0.2173	−1.6599	0.7094	0.064*	
C14A	0.17882 (11)	−1.4310 (6)	0.65937 (12)	0.0421 (8)	
O1B	0.14001 (7)	−0.4276 (4)	0.52608 (8)	0.0455 (6)	
O2B	0.06397 (7)	−0.5659 (4)	0.48193 (9)	0.0515 (7)	
O3B	0.09790 (8)	−1.3505 (4)	0.41681 (8)	0.0548 (7)	
O4B	0.17302 (8)	−1.2076 (5)	0.45538 (9)	0.0624 (8)	
C1B	0.14674 (10)	−0.6270 (5)	0.51492 (11)	0.0358 (7)	
C2B	0.11535 (10)	−0.7882 (5)	0.47961 (11)	0.0344 (7)	
C3B	0.07378 (11)	−0.7183 (6)	0.46274 (12)	0.0403 (8)	
C4B	0.04437 (11)	−0.8471 (7)	0.42133 (12)	0.0495 (9)	
H4B	0.0188	−0.7872	0.4062	0.059*	
C5B	0.05270 (11)	−1.0460 (7)	0.40446 (12)	0.0494 (9)	
H5B	0.033	−1.124	0.3779	0.059*	
C6B	0.09212 (11)	−1.1452 (6)	0.42663 (11)	0.0401 (8)	
C7B	0.12368 (10)	−0.9896 (5)	0.46217 (11)	0.0373 (8)	
C8B	0.16520 (11)	−1.0558 (6)	0.47708 (12)	0.0430 (9)	
C9B	0.19592 (11)	−0.9271 (6)	0.51772 (12)	0.0441 (9)	
C10B	0.23415 (12)	−1.0075 (7)	0.53751 (15)	0.0567 (10)	
H10B	0.2403	−1.1456	0.5252	0.068*	
C11B	0.26305 (13)	−0.8839 (8)	0.57533 (16)	0.0665 (12)	
H11B	0.2888	−0.9413	0.5893	0.08*	
C12B	0.25431 (12)	−0.6752 (8)	0.59277 (16)	0.0641 (11)	
H12B	0.2741	−0.5911	0.6184	0.077*	
C13B	0.21658 (12)	−0.5919 (7)	0.57241 (13)	0.0518 (10)	
H13B	0.2107	−0.4487	0.5837	0.062*	
C14B	0.18723 (10)	−0.7178 (6)	0.53537 (12)	0.0398 (8)	
O1C	−0.17305 (9)	−1.2788 (7)	0.17275 (13)	0.1042 (13)	
O2C	−0.17027 (10)	−1.6785 (6)	0.22155 (12)	0.0847 (10)	
O3CA	−0.01820 (11)	−1.5858 (18)	0.3207 (4)	0.114 (4)	0.643 (11)
O4CA	−0.02471 (16)	−1.1302 (12)	0.28814 (17)	0.083 (3)	0.643 (11)
O3CB	−0.0266 (3)	−1.4749 (18)	0.3371 (2)	0.060 (3)	0.357 (11)
O4CB	−0.02148 (18)	−1.2660 (18)	0.2633 (4)	0.057 (3)	0.357 (11)
C1C	−0.13826 (11)	−1.2674 (7)	0.19568 (13)	0.0503 (9)	
C2C	−0.11541 (11)	−1.4357 (6)	0.23584 (12)	0.0423 (8)	
C3C	−0.13602 (13)	−1.6384 (7)	0.24613 (14)	0.0512 (10)	
C4C	−0.11251 (14)	−1.7949 (6)	0.28644 (14)	0.0543 (10)	
H4C	−0.1248	−1.9216	0.2938	0.065*	
C5C	−0.07454 (14)	−1.7650 (7)	0.31302 (14)	0.0560 (11)	
H5C	−0.0608	−1.87	0.3387	0.067*	
C6C	−0.05317 (11)	−1.5716 (8)	0.30346 (14)	0.0646 (12)	
C7C	−0.07676 (11)	−1.4089 (6)	0.26261 (12)	0.0435 (8)	
C8C	−0.05391 (10)	−1.2096 (8)	0.25511 (15)	0.0687 (13)	
C9C	−0.07658 (11)	−1.0542 (6)	0.21240 (12)	0.0441 (8)	
C10C	−0.05745 (12)	−0.8738 (7)	0.20146 (13)	0.0510 (9)	
H10C	−0.0303	−0.8588	0.2196	0.061*	
C11C	−0.07745 (12)	−0.7176 (6)	0.16470 (12)	0.0475 (9)	
H11C	−0.0642	−0.5945	0.158	0.057*	
C12C	−0.11713 (12)	−0.7412 (7)	0.13742 (12)	0.0482 (9)	
H12C	−0.1309	−0.6332	0.1123	0.058*	
C13C	−0.13679 (11)	−0.9233 (7)	0.14693 (12)	0.0460 (9)	
H13C	−0.1638	−0.9409	0.1279	0.055*	
C14C	−0.11654 (10)	−1.0806 (6)	0.18478 (11)	0.0404 (8)	

### Atomic displacement parameters (Å^2^)

**Table:**

	*U*^11^	*U*^22^	*U*^33^	*U*^12^	*U*^13^	*U*^23^
O1A	0.064 (2)	0.0527 (15)	0.0550 (16)	0.0027 (14)	0.0230 (15)	0.0210 (13)
O2A	0.0585 (19)	0.0391 (13)	0.0710 (17)	0.0006 (12)	0.0376 (16)	0.0093 (12)
O3A	0.0491 (18)	0.0493 (14)	0.0499 (15)	0.0043 (12)	0.0187 (14)	0.0145 (12)
O4A	0.0461 (17)	0.0353 (12)	0.0601 (15)	0.0007 (11)	0.0288 (14)	0.0073 (11)
C1A	0.048 (2)	0.0338 (17)	0.0386 (18)	0.0020 (16)	0.0188 (18)	0.0004 (15)
C2A	0.038 (2)	0.0311 (16)	0.0392 (18)	0.0009 (14)	0.0186 (17)	−0.0014 (14)
C3A	0.046 (2)	0.0340 (17)	0.0440 (19)	−0.0005 (16)	0.0251 (19)	0.0023 (15)
C4A	0.036 (2)	0.0451 (19)	0.054 (2)	−0.0035 (16)	0.0219 (19)	0.0000 (17)
C5A	0.036 (2)	0.047 (2)	0.0455 (19)	0.0026 (16)	0.0164 (18)	−0.0012 (16)
C6A	0.043 (2)	0.0317 (16)	0.0379 (18)	0.0011 (15)	0.0188 (17)	0.0005 (14)
C7A	0.038 (2)	0.0305 (15)	0.0380 (17)	−0.0007 (14)	0.0202 (16)	−0.0017 (14)
C8A	0.039 (2)	0.0337 (16)	0.0403 (18)	−0.0012 (15)	0.0217 (17)	−0.0014 (14)
C9A	0.037 (2)	0.0367 (17)	0.0419 (19)	0.0014 (15)	0.0172 (17)	−0.0075 (15)
C10A	0.042 (2)	0.045 (2)	0.061 (2)	−0.0070 (18)	0.023 (2)	−0.0071 (18)
C11A	0.042 (3)	0.060 (2)	0.066 (3)	−0.005 (2)	0.024 (2)	−0.017 (2)
C12A	0.038 (3)	0.071 (3)	0.055 (2)	0.008 (2)	0.008 (2)	−0.015 (2)
C13A	0.049 (3)	0.054 (2)	0.044 (2)	0.006 (2)	0.013 (2)	−0.0029 (17)
C14A	0.042 (2)	0.0395 (18)	0.0385 (18)	0.0051 (16)	0.0151 (18)	−0.0031 (15)
O1B	0.0476 (16)	0.0359 (12)	0.0500 (14)	−0.0013 (11)	0.0215 (13)	−0.0080 (10)
O2B	0.0435 (17)	0.0439 (13)	0.0698 (17)	0.0030 (12)	0.0297 (15)	−0.0087 (12)
O3B	0.067 (2)	0.0400 (13)	0.0523 (15)	−0.0032 (13)	0.0252 (15)	−0.0118 (12)
O4B	0.055 (2)	0.0648 (17)	0.0672 (17)	0.0093 (14)	0.0297 (16)	−0.0172 (14)
C1B	0.040 (2)	0.0297 (16)	0.0381 (17)	0.0000 (15)	0.0194 (17)	−0.0005 (14)
C2B	0.037 (2)	0.0295 (15)	0.0341 (16)	0.0015 (14)	0.0150 (16)	0.0013 (13)
C3B	0.039 (2)	0.0343 (17)	0.0458 (19)	−0.0006 (15)	0.0189 (18)	0.0012 (15)
C4B	0.035 (2)	0.058 (2)	0.049 (2)	0.0014 (18)	0.0156 (19)	0.0018 (18)
C5B	0.042 (2)	0.055 (2)	0.042 (2)	−0.0069 (18)	0.0130 (19)	−0.0068 (17)
C6B	0.050 (2)	0.0354 (17)	0.0322 (17)	−0.0009 (16)	0.0184 (18)	−0.0029 (14)
C7B	0.041 (2)	0.0311 (16)	0.0363 (17)	0.0028 (15)	0.0163 (17)	0.0004 (14)
C8B	0.046 (2)	0.0375 (17)	0.046 (2)	0.0062 (16)	0.0230 (19)	0.0017 (16)
C9B	0.038 (2)	0.0406 (18)	0.051 (2)	−0.0007 (16)	0.0187 (19)	0.0008 (16)
C10B	0.043 (3)	0.056 (2)	0.071 (3)	0.0036 (19)	0.028 (2)	−0.005 (2)
C11B	0.036 (3)	0.075 (3)	0.081 (3)	0.004 (2)	0.023 (2)	−0.002 (2)
C12B	0.038 (3)	0.071 (3)	0.075 (3)	−0.013 (2)	0.022 (2)	−0.018 (2)
C13B	0.040 (2)	0.052 (2)	0.060 (2)	−0.0069 (18)	0.023 (2)	−0.0106 (18)
C14B	0.037 (2)	0.0358 (17)	0.046 (2)	−0.0019 (15)	0.0195 (18)	−0.0012 (15)
O1C	0.0304 (19)	0.127 (3)	0.125 (3)	−0.0038 (19)	0.014 (2)	0.072 (2)
O2C	0.054 (2)	0.085 (2)	0.098 (2)	−0.0204 (18)	0.024 (2)	0.0192 (19)
O3CA	0.044 (4)	0.130 (7)	0.116 (6)	0.004 (4)	−0.001 (4)	0.079 (5)
O4CA	0.037 (3)	0.109 (5)	0.064 (4)	−0.017 (3)	−0.006 (3)	0.032 (3)
O3CB	0.059 (7)	0.069 (6)	0.031 (4)	0.000 (5)	0.007 (4)	0.000 (4)
O4CB	0.034 (5)	0.076 (6)	0.059 (6)	0.014 (4)	0.021 (4)	0.025 (5)
C1C	0.030 (2)	0.061 (2)	0.057 (2)	0.0000 (18)	0.019 (2)	0.0115 (18)
C2C	0.039 (2)	0.0420 (18)	0.047 (2)	−0.0010 (16)	0.0215 (19)	0.0023 (16)
C3C	0.050 (3)	0.048 (2)	0.059 (2)	−0.0053 (19)	0.029 (2)	−0.0063 (18)
C4C	0.072 (3)	0.0399 (19)	0.063 (3)	−0.007 (2)	0.042 (3)	0.0013 (18)
C5C	0.073 (3)	0.049 (2)	0.049 (2)	0.000 (2)	0.032 (2)	0.0093 (18)
C6C	0.053 (3)	0.071 (3)	0.049 (2)	−0.009 (2)	0.008 (2)	0.018 (2)
C7C	0.036 (2)	0.0484 (19)	0.0416 (19)	0.0000 (17)	0.0154 (18)	0.0040 (16)
C8C	0.027 (2)	0.087 (3)	0.077 (3)	−0.002 (2)	0.013 (2)	0.042 (3)
C9C	0.030 (2)	0.050 (2)	0.047 (2)	0.0017 (16)	0.0148 (18)	0.0090 (16)
C10C	0.036 (2)	0.060 (2)	0.048 (2)	−0.0013 (18)	0.0135 (19)	0.0099 (18)
C11C	0.049 (3)	0.049 (2)	0.052 (2)	−0.0012 (18)	0.030 (2)	0.0051 (17)
C12C	0.048 (3)	0.053 (2)	0.043 (2)	0.0105 (19)	0.023 (2)	0.0094 (17)
C13C	0.033 (2)	0.060 (2)	0.044 (2)	0.0055 (17)	0.0177 (18)	0.0074 (17)
C14C	0.034 (2)	0.0449 (19)	0.0409 (19)	0.0045 (16)	0.0173 (18)	0.0056 (15)

### Geometric parameters (Å, º)

**Table:**

O1A—C1A	1.219 (4)	C7B—C8B	1.507 (5)
O2A—C3A	1.217 (4)	C8B—C9B	1.470 (5)
O3A—C6A	1.214 (4)	C9B—C10B	1.390 (5)
O4A—C8A	1.215 (4)	C9B—C14B	1.394 (5)
C1A—C14A	1.480 (5)	C10B—C11B	1.383 (6)
C1A—C2A	1.498 (5)	C10B—H10B	0.94
C2A—C7A	1.346 (4)	C11B—C12B	1.389 (5)
C2A—C3A	1.501 (5)	C11B—H11B	0.94
C3A—C4A	1.453 (5)	C12B—C13B	1.378 (5)
C4A—C5A	1.326 (5)	C12B—H12B	0.94
C4A—H4A	0.94	C13B—C14B	1.383 (5)
C5A—C6A	1.465 (5)	C13B—H13B	0.94
C5A—H5A	0.94	O1C—C1C	1.202 (5)
C6A—C7A	1.494 (5)	O2C—C3C	1.209 (5)
C7A—C8A	1.503 (5)	O3CA—C6C	1.2116 (10)
C8A—C9A	1.473 (5)	O4CA—C8C	1.2125 (10)
C9A—C14A	1.390 (5)	O3CB—C6C	1.2105 (11)
C9A—C10A	1.401 (5)	O4CB—C8C	1.2115 (10)
C10A—C11A	1.372 (5)	C1C—C14C	1.482 (5)
C10A—H10A	0.94	C1C—C2C	1.493 (5)
C11A—C12A	1.387 (6)	C2C—C7C	1.346 (5)
C11A—H11A	0.94	C2C—C3C	1.504 (5)
C12A—C13A	1.379 (6)	C3C—C4C	1.462 (5)
C12A—H12A	0.94	C4C—C5C	1.325 (6)
C13A—C14A	1.390 (5)	C4C—H4C	0.94
C13A—H13A	0.94	C5C—C6C	1.475 (5)
O1B—C1B	1.216 (3)	C5C—H5C	0.94
O2B—C3B	1.213 (4)	C6C—C7C	1.493 (5)
O3B—C6B	1.215 (4)	C7C—C8C	1.506 (5)
O4B—C8B	1.225 (4)	C8C—C9C	1.502 (5)
C1B—C14B	1.484 (5)	C9C—C10C	1.388 (5)
C1B—C2B	1.499 (4)	C9C—C14C	1.390 (5)
C2B—C7B	1.350 (4)	C10C—C11C	1.368 (5)
C2B—C3B	1.502 (5)	C10C—H10C	0.94
C3B—C4B	1.465 (5)	C11C—C12C	1.379 (5)
C4B—C5B	1.328 (5)	C11C—H11C	0.94
C4B—H4B	0.94	C12C—C13C	1.385 (5)
C5B—C6B	1.462 (5)	C12C—H12C	0.94
C5B—H5B	0.94	C13C—C14C	1.395 (5)
C6B—C7B	1.487 (5)	C13C—H13C	0.94
			
O1A—C1A—C14A	121.3 (3)	C9B—C8B—C7B	117.7 (3)
O1A—C1A—C2A	121.5 (3)	C10B—C9B—C14B	119.6 (3)
C14A—C1A—C2A	117.0 (3)	C10B—C9B—C8B	120.0 (3)
C7A—C2A—C1A	121.4 (3)	C14B—C9B—C8B	120.3 (3)
C7A—C2A—C3A	119.8 (3)	C11B—C10B—C9B	119.8 (4)
C1A—C2A—C3A	118.7 (3)	C11B—C10B—H10B	120.1
O2A—C3A—C4A	120.8 (3)	C9B—C10B—H10B	120.1
O2A—C3A—C2A	121.9 (3)	C10B—C11B—C12B	120.4 (4)
C4A—C3A—C2A	117.3 (3)	C10B—C11B—H11B	119.8
C5A—C4A—C3A	121.5 (3)	C12B—C11B—H11B	119.8
C5A—C4A—H4A	119.3	C13B—C12B—C11B	119.8 (4)
C3A—C4A—H4A	119.3	C13B—C12B—H12B	120.1
C4A—C5A—C6A	121.3 (3)	C11B—C12B—H12B	120.1
C4A—C5A—H5A	119.4	C12B—C13B—C14B	120.4 (4)
C6A—C5A—H5A	119.4	C12B—C13B—H13B	119.8
O3A—C6A—C5A	119.8 (3)	C14B—C13B—H13B	119.8
O3A—C6A—C7A	123.3 (3)	C13B—C14B—C9B	120.0 (3)
C5A—C6A—C7A	116.8 (3)	C13B—C14B—C1B	119.1 (3)
C2A—C7A—C6A	120.2 (3)	C9B—C14B—C1B	120.9 (3)
C2A—C7A—C8A	121.0 (3)	O1C—C1C—C14C	120.2 (3)
C6A—C7A—C8A	118.7 (3)	O1C—C1C—C2C	122.3 (3)
O4A—C8A—C9A	121.4 (3)	C14C—C1C—C2C	117.6 (3)
O4A—C8A—C7A	121.4 (3)	C7C—C2C—C1C	120.9 (3)
C9A—C8A—C7A	117.1 (3)	C7C—C2C—C3C	119.9 (3)
C14A—C9A—C10A	119.1 (3)	C1C—C2C—C3C	119.3 (3)
C14A—C9A—C8A	121.3 (3)	O2C—C3C—C4C	120.5 (4)
C10A—C9A—C8A	119.6 (3)	O2C—C3C—C2C	122.3 (4)
C11A—C10A—C9A	120.3 (4)	C4C—C3C—C2C	117.2 (4)
C11A—C10A—H10A	119.9	C5C—C4C—C3C	122.6 (4)
C9A—C10A—H10A	119.9	C5C—C4C—H4C	118.7
C10A—C11A—C12A	120.3 (4)	C3C—C4C—H4C	118.7
C10A—C11A—H11A	119.9	C4C—C5C—C6C	121.8 (4)
C12A—C11A—H11A	119.9	C4C—C5C—H5C	119.1
C13A—C12A—C11A	120.1 (4)	C6C—C5C—H5C	119.1
C13A—C12A—H12A	119.9	O3CB—C6C—C5C	116.7 (6)
C11A—C12A—H12A	119.9	O3CA—C6C—C5C	119.6 (5)
C12A—C13A—C14A	119.9 (4)	O3CB—C6C—C7C	116.3 (6)
C12A—C13A—H13A	120.1	O3CA—C6C—C7C	121.7 (4)
C14A—C13A—H13A	120.1	C5C—C6C—C7C	116.3 (3)
C13A—C14A—C9A	120.3 (4)	C2C—C7C—C6C	122.2 (3)
C13A—C14A—C1A	119.4 (3)	C2C—C7C—C8C	123.3 (3)
C9A—C14A—C1A	120.2 (3)	C6C—C7C—C8C	114.4 (3)
O1B—C1B—C14B	120.4 (3)	O4CB—C8C—C9C	115.7 (5)
O1B—C1B—C2B	122.4 (3)	O4CA—C8C—C9C	120.4 (4)
C14B—C1B—C2B	117.1 (3)	O4CB—C8C—C7C	115.6 (5)
C7B—C2B—C1B	121.5 (3)	O4CA—C8C—C7C	119.7 (4)
C7B—C2B—C3B	119.8 (3)	C9C—C8C—C7C	115.3 (3)
C1B—C2B—C3B	118.6 (3)	C10C—C9C—C14C	119.4 (3)
O2B—C3B—C4B	120.0 (3)	C10C—C9C—C8C	119.2 (3)
O2B—C3B—C2B	123.3 (3)	C14C—C9C—C8C	121.3 (3)
C4B—C3B—C2B	116.7 (3)	C11C—C10C—C9C	121.0 (4)
C5B—C4B—C3B	122.1 (4)	C11C—C10C—H10C	119.5
C5B—C4B—H4B	118.9	C9C—C10C—H10C	119.5
C3B—C4B—H4B	118.9	C10C—C11C—C12C	119.9 (3)
C4B—C5B—C6B	120.9 (3)	C10C—C11C—H11C	120
C4B—C5B—H5B	119.5	C12C—C11C—H11C	120
C6B—C5B—H5B	119.5	C11C—C12C—C13C	120.2 (3)
O3B—C6B—C5B	120.0 (3)	C11C—C12C—H12C	119.9
O3B—C6B—C7B	122.6 (3)	C13C—C12C—H12C	119.9
C5B—C6B—C7B	117.4 (3)	C12C—C13C—C14C	120.0 (3)
C2B—C7B—C6B	120.7 (3)	C12C—C13C—H13C	120
C2B—C7B—C8B	120.5 (3)	C14C—C13C—H13C	120
C6B—C7B—C8B	118.7 (3)	C9C—C14C—C13C	119.4 (3)
O4B—C8B—C9B	121.3 (3)	C9C—C14C—C1C	121.3 (3)
O4B—C8B—C7B	121.0 (3)	C13C—C14C—C1C	119.2 (3)
			
O1A—C1A—C2A—C7A	164.1 (3)	C7B—C8B—C9B—C10B	169.7 (3)
C14A—C1A—C2A—C7A	−12.3 (4)	O4B—C8B—C9B—C14B	165.3 (3)
O1A—C1A—C2A—C3A	−12.5 (4)	C7B—C8B—C9B—C14B	−13.1 (5)
C14A—C1A—C2A—C3A	171.1 (3)	C14B—C9B—C10B—C11B	1.9 (6)
C7A—C2A—C3A—O2A	166.1 (3)	C8B—C9B—C10B—C11B	179.1 (3)
C1A—C2A—C3A—O2A	−17.2 (4)	C9B—C10B—C11B—C12B	−2.0 (6)
C7A—C2A—C3A—C4A	−13.1 (4)	C10B—C11B—C12B—C13B	0.4 (6)
C1A—C2A—C3A—C4A	163.5 (3)	C11B—C12B—C13B—C14B	1.4 (6)
O2A—C3A—C4A—C5A	−166.9 (3)	C12B—C13B—C14B—C9B	−1.5 (5)
C2A—C3A—C4A—C5A	12.4 (5)	C12B—C13B—C14B—C1B	178.5 (3)
C3A—C4A—C5A—C6A	2.2 (5)	C10B—C9B—C14B—C13B	−0.1 (5)
C4A—C5A—C6A—O3A	163.5 (3)	C8B—C9B—C14B—C13B	−177.3 (3)
C4A—C5A—C6A—C7A	−15.7 (5)	C10B—C9B—C14B—C1B	179.8 (3)
C1A—C2A—C7A—C6A	−176.9 (3)	C8B—C9B—C14B—C1B	2.7 (5)
C3A—C2A—C7A—C6A	−0.4 (4)	O1B—C1B—C14B—C13B	10.0 (5)
C1A—C2A—C7A—C8A	1.0 (4)	C2B—C1B—C14B—C13B	−171.5 (3)
C3A—C2A—C7A—C8A	177.5 (3)	O1B—C1B—C14B—C9B	−170.0 (3)
O3A—C6A—C7A—C2A	−164.6 (3)	C2B—C1B—C14B—C9B	8.5 (4)
C5A—C6A—C7A—C2A	14.5 (4)	O1C—C1C—C2C—C7C	−175.9 (4)
O3A—C6A—C7A—C8A	17.4 (4)	C14C—C1C—C2C—C7C	3.6 (5)
C5A—C6A—C7A—C8A	−163.4 (3)	O1C—C1C—C2C—C3C	4.4 (6)
C2A—C7A—C8A—O4A	−169.0 (3)	C14C—C1C—C2C—C3C	−176.0 (3)
C6A—C7A—C8A—O4A	8.9 (4)	C7C—C2C—C3C—O2C	−175.7 (4)
C2A—C7A—C8A—C9A	9.1 (4)	C1C—C2C—C3C—O2C	3.9 (5)
C6A—C7A—C8A—C9A	−173.0 (3)	C7C—C2C—C3C—C4C	1.7 (5)
O4A—C8A—C9A—C14A	170.5 (3)	C1C—C2C—C3C—C4C	−178.7 (3)
C7A—C8A—C9A—C14A	−7.7 (4)	O2C—C3C—C4C—C5C	176.7 (4)
O4A—C8A—C9A—C10A	−8.7 (5)	C2C—C3C—C4C—C5C	−0.7 (5)
C7A—C8A—C9A—C10A	173.2 (3)	C3C—C4C—C5C—C6C	−0.5 (6)
C14A—C9A—C10A—C11A	1.7 (5)	C4C—C5C—C6C—O3CB	144.1 (7)
C8A—C9A—C10A—C11A	−179.1 (3)	C4C—C5C—C6C—O3CA	−162.1 (9)
C9A—C10A—C11A—C12A	−0.3 (5)	C4C—C5C—C6C—C7C	0.9 (6)
C10A—C11A—C12A—C13A	−2.0 (6)	C1C—C2C—C7C—C6C	179.0 (3)
C11A—C12A—C13A—C14A	2.7 (6)	C3C—C2C—C7C—C6C	−1.3 (5)
C12A—C13A—C14A—C9A	−1.2 (5)	C1C—C2C—C7C—C8C	1.1 (6)
C12A—C13A—C14A—C1A	−177.7 (3)	C3C—C2C—C7C—C8C	−179.3 (3)
C10A—C9A—C14A—C13A	−1.0 (5)	O3CB—C6C—C7C—C2C	−143.3 (7)
C8A—C9A—C14A—C13A	179.8 (3)	O3CA—C6C—C7C—C2C	162.7 (9)
C10A—C9A—C14A—C1A	175.5 (3)	C5C—C6C—C7C—C2C	0.1 (6)
C8A—C9A—C14A—C1A	−3.7 (5)	O3CB—C6C—C7C—C8C	34.9 (8)
O1A—C1A—C14A—C13A	13.7 (5)	O3CA—C6C—C7C—C8C	−19.2 (10)
C2A—C1A—C14A—C13A	−169.9 (3)	C5C—C6C—C7C—C8C	178.2 (4)
O1A—C1A—C14A—C9A	−162.8 (3)	C2C—C7C—C8C—O4CB	−145.1 (7)
C2A—C1A—C14A—C9A	13.6 (4)	C6C—C7C—C8C—O4CB	36.8 (8)
O1B—C1B—C2B—C7B	169.3 (3)	C2C—C7C—C8C—O4CA	150.2 (6)
C14B—C1B—C2B—C7B	−9.2 (4)	C6C—C7C—C8C—O4CA	−27.9 (7)
O1B—C1B—C2B—C3B	−9.3 (4)	C2C—C7C—C8C—C9C	−5.7 (6)
C14B—C1B—C2B—C3B	172.3 (3)	C6C—C7C—C8C—C9C	176.2 (4)
C7B—C2B—C3B—O2B	167.5 (3)	O4CB—C8C—C9C—C10C	−37.6 (8)
C1B—C2B—C3B—O2B	−13.9 (5)	O4CA—C8C—C9C—C10C	27.4 (8)
C7B—C2B—C3B—C4B	−11.6 (4)	C7C—C8C—C9C—C10C	−176.9 (3)
C1B—C2B—C3B—C4B	167.0 (3)	O4CB—C8C—C9C—C14C	145.2 (7)
O2B—C3B—C4B—C5B	−166.9 (3)	O4CA—C8C—C9C—C14C	−149.8 (6)
C2B—C3B—C4B—C5B	12.3 (5)	C7C—C8C—C9C—C14C	5.9 (6)
C3B—C4B—C5B—C6B	−0.3 (5)	C14C—C9C—C10C—C11C	1.7 (5)
C4B—C5B—C6B—O3B	165.7 (3)	C8C—C9C—C10C—C11C	−175.6 (4)
C4B—C5B—C6B—C7B	−12.1 (5)	C9C—C10C—C11C—C12C	−1.1 (5)
C1B—C2B—C7B—C6B	−179.1 (3)	C10C—C11C—C12C—C13C	−0.4 (5)
C3B—C2B—C7B—C6B	−0.5 (4)	C11C—C12C—C13C—C14C	1.3 (5)
C1B—C2B—C7B—C8B	−1.4 (4)	C10C—C9C—C14C—C13C	−0.8 (5)
C3B—C2B—C7B—C8B	177.2 (3)	C8C—C9C—C14C—C13C	176.4 (4)
O3B—C6B—C7B—C2B	−165.3 (3)	C10C—C9C—C14C—C1C	−178.8 (3)
C5B—C6B—C7B—C2B	12.5 (4)	C8C—C9C—C14C—C1C	−1.6 (5)
O3B—C6B—C7B—C8B	16.9 (5)	C12C—C13C—C14C—C9C	−0.7 (5)
C5B—C6B—C7B—C8B	−165.3 (3)	C12C—C13C—C14C—C1C	177.4 (3)
C2B—C7B—C8B—O4B	−165.8 (3)	O1C—C1C—C14C—C9C	176.2 (4)
C6B—C7B—C8B—O4B	12.0 (5)	C2C—C1C—C14C—C9C	−3.3 (5)
C2B—C7B—C8B—C9B	12.6 (4)	O1C—C1C—C14C—C13C	−1.8 (6)
C6B—C7B—C8B—C9B	−169.6 (3)	C2C—C1C—C14C—C13C	178.7 (3)
O4B—C8B—C9B—C10B	−11.9 (5)		

### Hydrogen-bond geometry (Å, º)

**Table:**

*D*—H···*A*	*D*—H	H···*A*	*D*···*A*	*D*—H···*A*
C4*C*—H4*C*···O1*A*^i^	0.94	2.45	3.373 (5)	169
C5*C*—H5*C*···O2*A*^i^	0.94	2.33	3.112 (5)	140
C11*A*—H11*A*···O1*C*^ii^	0.94	2.55	3.262 (7)	133
C11*C*—H11*C*···O3*B*^iii^	0.94	2.52	3.113 (4)	121
C12*C*—H12*C*···O3*B*^iii^	0.94	2.49	3.095 (5)	122

Symmetry codes: (i) −*x*, −*y*−4, −*z*+1; (ii) *x*+1/2, −*y*−5/2, *z*+1/2; (iii) −*x*, *y*+1, −*z*+1/2.
